# An Inertial-Based Wearable System for Monitoring Vital Signs during Sleep

**DOI:** 10.3390/s24134139

**Published:** 2024-06-26

**Authors:** Spyridon Kontaxis, Foivos Kanellos, Adamantios Ntanis, Nicholas Kostikis, Spyridon Konitsiotis, George Rigas

**Affiliations:** 1 PD Neurotechnology Ltd., 45500 Ioannina, Greeceg.rigas@pdneurotechnology.com (G.R.); 2 Department of Physiology, Faculty of Medicine, School of Health Sciences, University of Ioannina, 45110 Ioannina, Greece; 3 University Hospital of Ioannina and Faculty of Medicine, School of Health Sciences, University of Ioannina, 45110 Ioannina, Greece; skonitso@gmail.com

**Keywords:** respiratory rate, heart rate, sleep disorders, polysomnography, seismocardiography, gyrocardiography

## Abstract

This study explores the feasibility of a wearable system to monitor vital signs during sleep. The system incorporates five inertial measurement units (IMUs) located on the waist, the arms, and the legs. To evaluate the performance of a novel framework, twenty-three participants underwent a sleep study, and vital signs, including respiratory rate (RR) and heart rate (HR), were monitored via polysomnography (PSG). The dataset comprises individuals with varying severity of sleep-disordered breathing (SDB). Using a single IMU sensor positioned at the waist, strong correlations of more than 0.95 with the PSG-derived vital signs were obtained. Low inter-participant mean absolute errors of about 0.66 breaths/min and 1.32 beats/min were achieved, for RR and HR, respectively. The percentage of data available for analysis, representing the time coverage, was 98.3% for RR estimation and 78.3% for HR estimation. Nevertheless, the fusion of data from IMUs positioned at the arms and legs enhanced the inter-participant time coverage of HR estimation by over 15%. These findings imply that the proposed methodology can be used for vital sign monitoring during sleep, paving the way for a comprehensive understanding of sleep quality in individuals with SDB.

## 1. Introduction

Monitoring of vital signs provides valuable insights into a person’s overall health. Substantial evidence indicates that the prediction of potentially severe adverse clinical events, such as cardiac arrest [1] and respiratory failure [2], relies on the identification of abnormalities in vital signs. The respiratory rate (RR) and the heart rate (HR) are considered to be the most significant vital signs in assessing physiological deterioration [3] and determining the need for admission to the Intensive Care Unit (ICU) [4]. Furthermore, the continuous monitoring allows healthcare professionals to assess disease trends and treatment outcomes [5], as well as to reduce the occurrence of post-surgical complications [6]. For decades, assessing vital signs required the use of cumbersome and expensive equipment, causing patient discomfort and limiting their use in certain applications such as ambulatory monitoring or home care settings [7].

In the era of wearables, a wide range of sensors have become essential for non-intrusive remote patient monitoring [8]. The main technologies utilized for continuous monitoring of cardiorespiratory parameters include the electrocardiogram (ECG) [9,10,11], the photoplethysmogram [12,13,14], and the respiratory inductance plethysmography [15]. The aforementioned signals are considered the gold standard in monitoring vital signs and usually they are collected through the use of patches, textile sensors, chest straps, and wristbands, with the latter gaining greater popularity thanks to the commercial progress of fitness trackers [16]. Indirect measurement of both cardiac and respiratory changes can also be achieved by monitoring body motion. Body movements due to the ejection of blood into the great vessels with each heartbeat and breathing have been recorded with multiple types of sensors, including both wearables and non-wearables, which are capable of measuring displacement, acceleration, or angular velocity [17]. For instance, the ballistocardiogram (BCG) records the displacement of the body’s center of mass (resulting from blood circulation and heart displacement) throughout the cardiac cycle [18]. BCG is used mainly in sleep assessment to monitor vital signs, with sensors integrated into objects like mattresses, pillows, and beds [19]. In the case of wearable sensors, the seismocardiogram (SCG) and the gyrocardiogram (GCG) capture the compression waves of the heart contraction by attaching an accelerometer and a gyroscope, respectively, to various body locations (e.g., chest, arms, head, or feet) [20,21]. When inertial sensors are placed near the chest, the accelerations and angular velocities they measure are also affected by the expansion and contraction of the lungs, thus allowing breathing monitoring [22].

The initial attempt to track human vital signs via inertial sensors was conducted in 2006 by Reinvuo et al. [23], who utilized a sensor belt equipped with a dual-axis accelerometer to measure RR. In 2013, Bieber et al. [24] achieved HR monitoring when a wrist-worn three-axis (3D) accelerometer was positioned near the chest. Since then, considerable research has been conducted to explore the potential use of SCG and GCG to monitor vital signs. The majority of studies have thoroughly explored the effectiveness of inertial sensors in situations with minimal motion, such as when individuals are in reclined or seated postures [25,26,27,28,29,30], as well as during sleep monitoring [31,32,33,34,35]. However, there is a limited body of research that has examined the reliability of inertial sensors in ambulatory recordings [36,37,38,39], and among clinical populations, such as ICU patients following hospitalization [40] and individuals with sleep-disordered breathing (SDB) [41,42].

Apart from analyzing sensor type and positioning [43,44,45], there has been significant research conducted to create methodological frameworks aimed at improving the precision of estimating cardiorespiratory parameters. The key concepts of the most widely-used methodologies to increase the signal-to-noise ratio (SNR) involve choosing the optimal axis, combining axes, or performing a subsequent fusion of estimated rates. Simple axis selection strategies include choosing the dorso-ventral axis, which exhibits the strongest cardiorespiratory modulations [30,42], or the one with the highest auto-correlation [41] or frequency power [32] within the expected modulation range. Fusion methods involve various techniques, ranging from basic approaches like calculating the magnitude [35] or averaging normalized waveforms [36] to employing more advanced signal decomposition methods, such as principal component analysis (PCA) and singular spectrum analysis, on axes [29,31] or quaternions [25]. Various techniques for merging estimated rates from different sensors or axes comprise the utilization of a Kalman filter [37] or a weighted average, considering factors such as the amplitude of the maximum spectral peak [28,34], and the similarity with previous estimates [26].

In this study, the feasibility of an inertial-based wearable system to monitor vital signs during sleep is investigated. The system incorporates five inertial measurement units (IMUs) located on the waist, the arms, and the legs. Only a few studies have employed multiple IMUs, but mostly on the torso, to mitigate movement artifacts in ambulatory recordings. This work presents a novel signal processing pipeline that allows the fusion of the most informative axes in a dynamic manner. The methodology is founded upon the application of an SNR-dependent spectral average that has been previously employed in ECG-derived respiration signals [10,13,46]. In this study, a weighted-function is used to amplify SNR for spectra with dominant rate close to previous estimations. The efficacy of the suggested approach is assessed in terms of error and percentage of data discarded due to low SNR in individuals with varying severity of SDB. A comparative analysis with prior research is undertaken, considering factors such as the presence of SDB, the number of sensors and their placement, as well as the fusion methodologies (both intra- and inter-IMU) employed to derive vital signs.

## 2. Materials and Methods

### 2.1. Dataset

A total of 23 participants underwent a sleep study in order to examine the feasibility of an IMU-based estimation of vital signs. The studied population is composed of individuals with varying severity of apnea–hypopnea index (AHI), defined as the total number of apneas and hypopneas per hour of sleep. Participants with an AHI of less than 5 events per hour are classified as individuals without obstructive sleep apnea (OSA). An AHI of at least 5 events per hour, but fewer than 15, is the threshold to diagnose mild OSA. Patients with moderate OSA experience 15 to 30 episodes per hour, while in severe OSA, a person will experience 30 or more episodes in an hour. Eligible candidates were adults and were expected to be free from other significant medical conditions that might impact their suitability for the study. The demographic characteristics of the eligible subjects are displayed in Table 1.

The participants completed an overnight polysomnography (PSG) at the Centre of Sleep Disorders located at “Evangelismos” Hospital in Athens, Greece. Vital signs, including RR, $f_{\mathrm{RR}}\left(k\right)$, and HR, $f_{\mathrm{HR}}\left(k\right)$, were monitored via PSG at a sampling period of Δk=6 s. Additionally, a body-worn IMU system (see description in Section 2.2) was employed to estimate these vital indicators.

### 2.2. IMU-Based Wearable System

**Figure 1:**
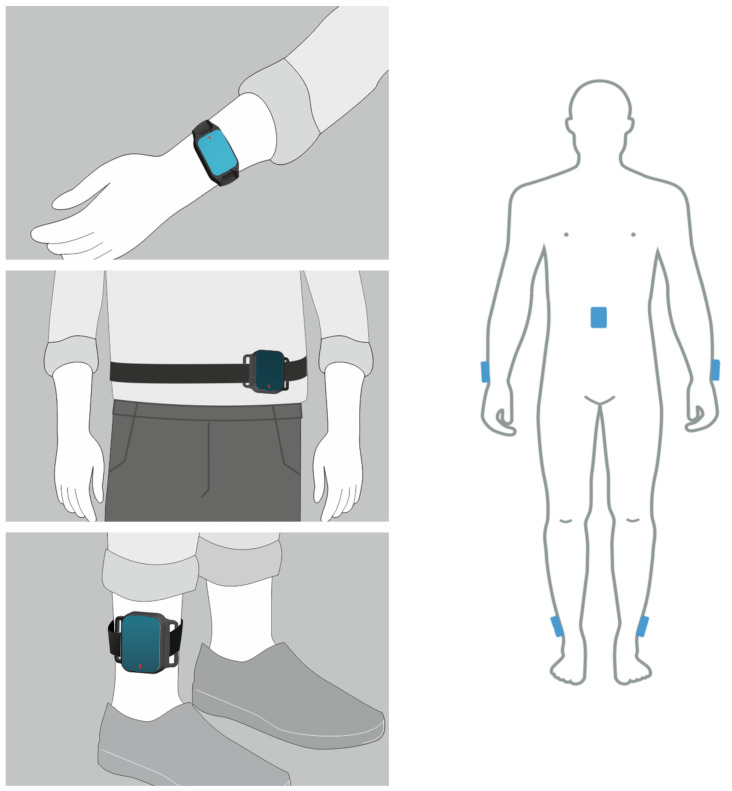
Placement of the PDMonitor^®^ sensors on the body. The sensors on the waist and the lateral compartments of the legs are attached with Velcro bands and StrapFrames, while wristbands are used to attach the sensors worn on the posterior compartment of the forearm.

The body-worn IMU system that was employed to collect motion data was the PDMonitor^®^ system. The PDMonitor^®^ is a class IIa CE-marked medical device, developed by PD Neurotechnology Ltd., and was designed for continuous home monitoring of patients who have been diagnosed with Parkinson’s Disease (PD). The system consists of five 9-degree IMU sensors that collect motion data at 59.5 Hz sampling frequency. Each IMU sensor is an LSM9DS1 from ST Microelectronics with linear acceleration full scale of ±4 g, a magnetic field full scale of ±4 gauss, and an angular rate of ±500 dps. The monitoring devices were placed on the waist, wrists, and ankles, as illustrated in Figure 1. More details about PDMonitor^®^ can be found in [47,48].

### 2.3. IMU-Derived Vital Signs

**Figure 2:**
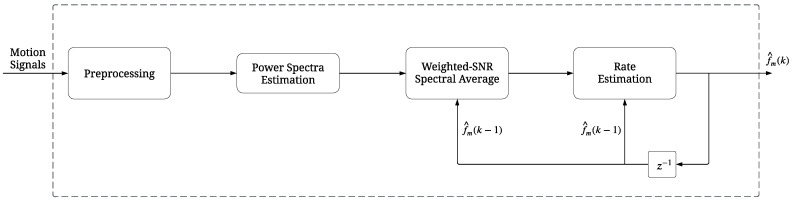
Block diagram for deriving vital signs, ${\hat{f}}_{m}\left(k\right)$, from motion signals.

In this study, a novel signal processing framework for estimating the dominant rate ${\hat{f}}_{m}\left(k\right)$ associated with respiratory (m=RR) and cardiac (m=HR) modulation from IMU-based spectra is presented. The framework employed in this study relies exclusively on data obtained from 3D accelerometers and gyroscopes (J=6 axes), since magnetic field variations in unstructured environments can reduce the quality of body motion data [43]. First, the *J* motion signals undergo band-pass filtering in two frequency ranges associated with respiratory ([0.08, 0.6] Hz) and cardiac ([0.7, 1.7] Hz) activity during sleep. For each motion signal, power spectrum estimation is carried out using the Welch periodogram every Δk s (*k*:th occurrence) in $T_{k}=30$ s symmetrical windows. Utilizing the position of the maximum peak to determine the dominant rate may show considerable fluctuations across spectra and axes due to the presence of noise and motion artifacts. In order to reduce the variance, only spectra exhibiting a high SNR should be taken into account. Such spectra can be identified by computing the power in a narrow symmetrical band around the maximum peak (typically 2δ=0.2 Hz). The distance between the maximum peak and previous rate position is used as a weight to amplify SNR for spectra with a dominant rate that deviates less than δ Hz. For the *k*:th instance, considering stationarity of the dominant rate in an interval of length $T_{k}+\left(L-1\right)\cdot \Delta k$ s, up to L=6 spectra can be averaged [10]. Only spectra with SNR>0.5 participate in the calculation of the averaged spectrum. Note that the resulting spectrum is an average from zero up to L×J power spectra. The dominant rate is defined based on the position of the local maximum that is located closer to the previous estimation (${\hat{f}}_{m}\left(k-1\right)$). The block diagram for deriving vital signs from motion signals is illustrated in Figure 2.

### 2.4. Performance Evaluation

The feasibility of the proposed methodology to estimate the RR and the HR from an IMU-based wearable system is evaluated by assessing the Mean Absolute Error (MAE) for each participant in comparison to the reference vital signs obtained through PSG. The calculation of the time coverage ($T_{C}$), which refers to the proportion of time intervals with high-quality estimations, is also performed for each participant in order to facilitate performance comparison. The quantification of the bivariate association between the estimated and reference vital signs is accomplished by employing Spearman’s Correlation (ρ) for all pairs of measurements. Mean of the differences (*d*) and limits of agreement (LoAs) from −1.96·SD to +1.96·SD are also calculated using all pairs of measurements. The statistical significance of the bias was assessed by conducting a one-sample *t*-test for *d* versus a zero-mean distribution.

First, performance evaluation, including correlation and Bland–Altman plots, as well as the median and interquartile range (IQR) of the MAE and $T_{C}$ metrics, was carried out for the IMU sensor placed on the waist (${\mathrm{IMU}}_{W}$). Furthermore, the influence of the AHI on the median MAE and $T_{C}$ metrics was quantified with Spearman’s correlation. Then, a Kruskal–Wallis nonparametric one-way analysis of variance was conducted to determine differences in median MAE and $T_{C}$ between various combinations of IMU_W_ with IMUs placed on the arms (IMU_A_) and/or the legs (IMU_L_). The combinations being analyzed are the one with the IMU_W_ and IMU_A_ (IMU_WA_), the one with the IMU_W_ and IMU_L_ (IMU_WL_), and the one with all sensors together (IMU_WAL_). To identify which IMU combination originates from distinct distributions, multi-comparison tests (post hoc analysis) with Bonferroni correction were carried out using the Wilcoxon signed rank test. In this study, the significance threshold was set to (p<0.05).

## 3. Results

**Figure 3:**
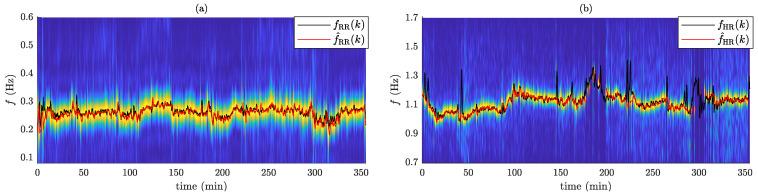
Time-frequency maps for the weigted SNR averaged spectra. (**a**) RR estimation and (**b**) HR estimation. The reference rate and the estimated rate are displayed with black solid and red solid lines, respectively.

**Figure 4:**
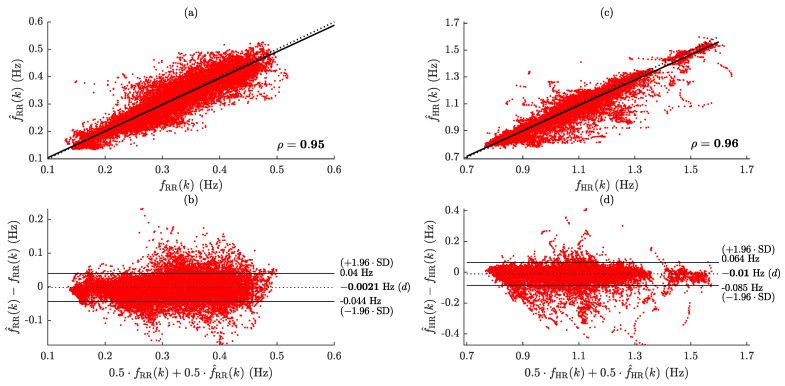
Evaluation of IMU_W_ performance using correlation and Bland–Altman plots. (**a**,**b**) RR estimation, and (**c**,**d**) HR estimation. In bold are marked statistically significant correlation and bias values (p<0.05).

Figure 3 shows an example of RR and HR estimation for a subject wearing the waist IMU sensor. The performance metrics acquired for the waist-worn IMU sensor (${\mathrm{IMU}}_{W}$) are presented in Table 2. Regarding the evaluation of the error, MAE = 0.011 (0.008) Hz, i.e., 0.66 (0.48) min^−1^, and MAE = 0.022 (0.014) Hz, i.e., 1.32 (0.84) min^−1^, were obtained for RR and HR, respectively. The time coverage for RR estimation was high in almost all subjects, with a value of $T_{C}=98.3\;\left(3.6\right)\%$, while HR estimation was not feasible in two out of twenty-three participants, resulting in a time coverage of $T_{C}=78.3\;\left(26.2\right)\%$. A strong correlation was found between the AHI and the error in estimating RR (ρ=0.69), as well as with the time coverage of HR estimation (ρ=−0.52). Correlation and Bland–Altman plots for evaluating ${\mathrm{IMU}}_{W}$ performance are illustrated in Figure 4. Results show that the estimations exhibit an excellent correlation with the references, yielding ρ=0.95 (RR) and ρ=0.96 (HR). The present method also yielded accurate results in terms of error. A statistically significant bias of less than −0.13 min^−1^ and −0.6 min^−1^ were found for RR and HR, respectively. The LoAs for RR span from −0.044 to 0.040 Hz (−2.64 to 2.40 min^−1^), while for HR, they extend from −0.085 to 0.064 Hz (−5.10 to 3.84 min^−1^).

Table 3 provides a summary of performance metrics from previous research in sleep studies. The vast majority of the studies utilized 3D accelerometers, with only one study integrating the accelerometer with a 3D gyroscope. Most research provides correlation and MAE values, but only a few of them include information about the portion of data that were discarded. Most studies found correlation coefficients higher than ρ>0.9 for both RR and HR. In terms of error, various studies have documented an RR that exceeds an MAE of 1.5 breaths/min. A particular study involving three individuals revealed a mean error of 0.95 beats/min for HR, though with an SD close to 3.5 beats/min. Time coverage metrics were only reported in studies focused on deploying sensors on the wrist. These metrics varied, with a range of 25.7% in extensive clinical populations (AHI>5) to more than 48.3% in smaller datasets consisting of individuals without OSA (AHI<5).

**Figure 5:**
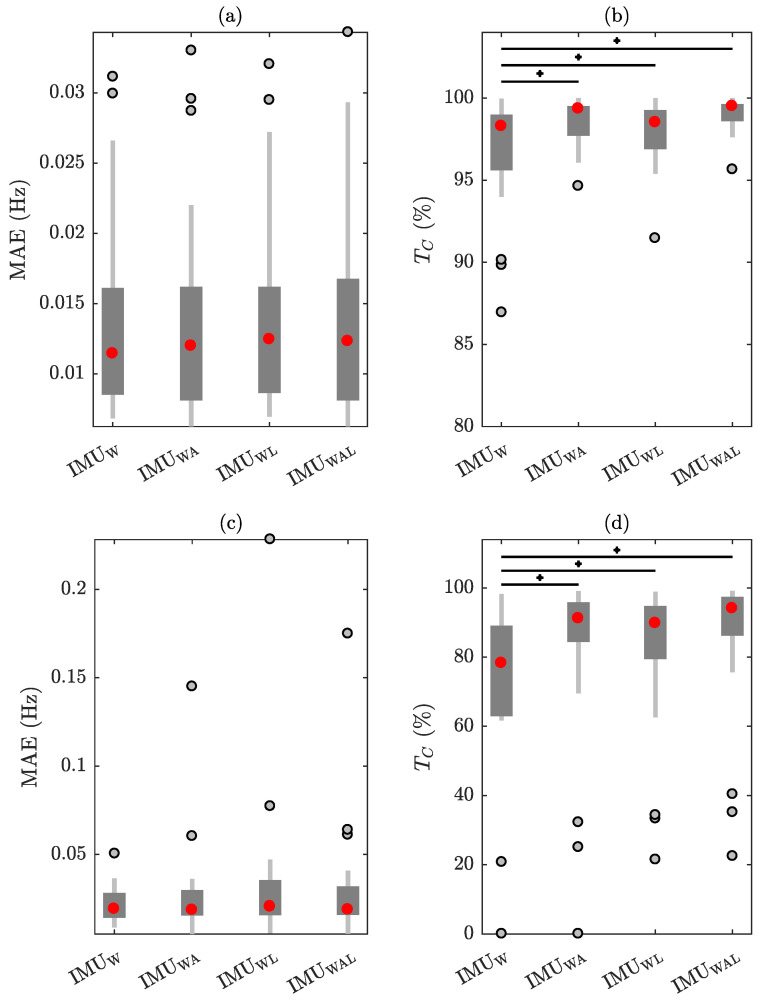
The effect of combining different IMUs placed on the waist (W), arms (A), and legs (L) on the performance metrics. (**a**,**b**) RR estimation, (**c**,**d**) HR estimation. Statistical differences of performance metrics between ${\mathrm{IMU}}_{W}$ and ${\mathrm{IMU}}_{\mathrm{WA}}$, ${\mathrm{IMU}}_{\mathrm{WL}}$, or ${\mathrm{IMU}}_{\mathrm{WAL}}$ are marked with an asterisk (p<0.05).

The influence of integrating information from several IMUs on the performance metrics is depicted in Figure 5. The results indicate that incorporating more IMUs positioned at the extremities does not result in a significant change in the MAE. However, there is a considerable increase in the time coverage for both RR and HR. More precisely, when all sensors were used, the estimation of RR attained a value of $T_{C}=99.5\;\left(1.0\right)\%$, while the estimation of HR reached $T_{C}=94.1\;\left(11.2\right)\%$. Nevertheless, it is evident from the presence of certain outliers in MAE and $T_{C}$ distributions that incorporate additional IMUs may not always yield advantageous results for all individuals. This is due the fact that IMUs positioned at the extremities are more susceptible to movement artifacts than the IMU placed around the waist.

## 4. Discussion

This study examines the feasibility of a wearable system using IMUs to track vital signs while a person is asleep. The system integrates five IMUs positioned on the waist, arms, and legs. The study introduces a new weighted function designed to enhance SNR for spectra with a dominant rate close to previous estimations. This approach allows the integration of information across multiple axes and IMU sensors. The effectiveness of this approach is evaluated in individuals with varying severity of SDB.

Performance metrics for previous works involving recordings during sleep provide valuable insights into the feasibility and efficiency of the current methodology. For a comprehensive evaluation of its effectiveness, such metrics are summarized in Table 3. Results indicate that employing a sole IMU sensor positioned at the waist demonstrates robust performance, as evidenced by its high correlation (ρ≥0.95) with reference rates derived from PSG. Similar results have been reported in most of the previous studies [32,33,41]. Regarding MAE, the current approach, i.e., $\mathrm{MAE}=0.66\;\left(0.48\right)\;\min^{-1}$, yielded an improvement of nearly 1 breath/min in estimating RR compared to [35,42]. However, there was only one study with a limited number of participants that reported a slightly better mean error for HR [34]. Nonetheless, the standard deviation was approximately 3.5 beats/min, compared to the 0.84 beats/min achieved in this study, i.e., $\mathrm{MAE}=1.32\;\left(0.84\right)\;\min^{-1}$. The estimation of RR showed a high time coverage in the majority of subjects ($T_{C}=98.3\%$), in contrast to HR estimation, which achieved lower values ($T_{C}=78.3\%$). Much lower time coverage values ($T_{C}<50\%$) were reported in previous studies that analyzed healthy individuals [32] and patients with SDB [41], for both RR and HR estimation.

It should be noted that performance can be influenced by medical conditions, including the severity of SDB. Results showed that AHI exhibits a strong positive correlation with the error in estimating RR (ρ=0.69), and a negative association with the time coverage of HR estimation (ρ=−0.52). This implies that participants with higher AHIs have lower data quality, leading to an elevated MAE and a decrease in $T_{C}$. While data from SDB patients were examined in just two studies, only one of them documented an AHI of 5.8(4.2) [42]. This is the first study reporting a much higher AHI of 27.5(33.0). This implies that the proposed methodology can be used for vital sign monitoring during sleep in healthy subjects as well as in a clinical sample of subjects with SDB.

Besides clinical characteristics, sensor placement can also have an impact on the performance. An accurate estimation of vital signs requires the IMU to be placed near the chest [24,32]. For instance, HR monitoring with devices positioned at the extremities is constrained by limited time coverage [41]. On the contrary, estimating RR using IMUs placed in close proximity to the chest and abdomen could lead to similar results [42]. Note that the placement of IMUs on specific body parts may reduce their convenience for regular individual use [49]. For instance, the chest was found to be the least preferred location compared to waist, ankles, and wrists [50,51]. Although the waist might be the most kinematically stable location with minimal variation due to nocturnal movements, placing IMUs at the extremities could improve the performance. In this study, a significant rise in time coverage was found when combining IMUs placed on different body locations (Figure 5). In particular, the median $T_{C}$ was increased by over 15% when utilizing all IMUs for estimating HR.

Different methodologies have been employed to improve the estimation of vital signs during sleep. In [42], the authors selected the axis perpendicular to the chest surface, considering that participants were lying in a supine position. In [41], the axis with the highest auto-correlation peak was chosen. However, opting for a single axis may prove insufficient when the individual changes sleep positions during overnight recordings. On the contrary, by combining axes, a novel one-dimensional signal can be derived using fusion techniques such as calculating the magnitude [35] or employing PCA [31,33]. Nevertheless, if certain axes are contaminated by artifacts, the fusion of all axes in a non-dynamic fashion could potentially lower the SNR in the resulting signal. Time-frequency approaches to improve the accuracy of detecting the dominant rate were introduced in [32,34]. These approaches relied on spectral amplitude as a foundation, while this study introduced the incorporation of prior estimations to augment the available information for determining the dominant rate.

Another novelty of the current study is the use of multiple IMUs positioned at different body locations to enhance the effectiveness of monitoring vital signs. This innovative aspect extends beyond sleep research, since even in the limited studies employing multiple IMUs, the positioning of sensors was confined to the torso [38,39]. While in this study, cardiorespiratory-induced accelerations and angular velocities across the whole body are combined, in previous studies the additional sensors aimed mainly at acquiring a reference for rejecting motion artifacts in ambulatory recordings. However, the comparison of performance metrics during situations where the subjects were walking or performing activities is not straightforward. The performance can also exhibit considerable fluctuations even when dealing with recordings featuring minimal motion, owing to differences in body position [27].

One drawback of the current study is that the estimation of vital signs requires stationarity of the dominant rate in one-minute-long intervals. Although this duration is suitable for estimating RR, quantification of HR patterns, besides slow variations, is not feasible (Figure 3). Reducing the size of the analysis window will lead to increased MAE values [30]. On the other hand, the utilization of adaptive filtering incorporating the estimated trends of RR and HR, in conjunction with peak detection approaches, has the potential to improve resolution [38]. Another limitation is that the performance of IMU-derived vital signs during sleep may be altered in the case of comorbid medical conditions, including sleep-related movement disorders. For instance, the joint analysis of IMUs placed at the extremities can hinder the performance in the case of periodic limb movements. In this study, employing additional IMUs to the IMU positioned around the waist did not consistently produce favorable outcomes across all participants (Figure 5). Further analyses considering artifact rejection methods based on signal energy at the preprocessing stage and different thresholds for the lowest acceptable SNR in the spectral average operation should be conducted. Moreover, additional studies for assessing the usability and wearability of the current system in home settings should be conducted.

The findings of this study open up the opportunity to explore physiological variations in respiratory patterns and circadian rhythms during sleep, eliminating the need for PSG recordings. This information can be applied to evaluate the probability of experiencing SDB [52,53], construct indices that measure the overall sleep quality [54], and improve actigraphy-based features to better distinguish different sleep stages [32,55].

## 5. Conclusions

This study demonstrates the feasibility of monitoring RR and HR during sleep using an inertial-based wearable system. A novel weighted function was introduced to enhance SNR for spectra with a dominant rate close to previous estimations. The proposed methodological framework facilitates the integration of information across various axes and IMU sensors distributed across the entire body. Results indicate that employing a single IMU sensor placed at the waist exhibits strong performance, as indicated by its high correlation (ρ≥0.95) with reference vital signs obtained from PSG. Across all participants, mean absolute errors of 0.66 breaths/min and 1.32 beats/min were found for RR and HR, respectively. The estimation of RR achieved a high time coverage in the majority of subjects (98.3%), while HR estimation reached lower values (78.3%). Nevertheless, the latter improved by over 15% when combining IMUs positioned at the arms and the legs. The effectiveness of the suggested approach proves to be robust in subjects with varying severity of SDB.

## Figures and Tables

**Table 1:** Demographic data of the participants.

Aggregated Demographics	Values
Number of participants	23
Sex (Male/Female)	15/8
Age (years)	46.6 (14.9)
Body mass index (kg/m^2^)	29.5 (8.1)
AHI (events/hour)	27.5 (33.0)
No OSA (AHI<5)	6
Mild OSA (5≤AHI<15)	9
Moderate OSA (15≤AHI<30)	1
Severe OSA (AHI≥30)	7

Values are denoted as numbers or mean (SD); AHI: apnea–hypopnea index; OSA: obstructive sleep apnea.

**Table 2:** Performance metrics acquired for IMU_W_.

	RR		HR	
**ID**	**MAE (Hz)**	$T_{C}\;\left(\%\right)$	**MAE (Hz)**	$T_{C}\;\left(\%\right)$
1	0.015	94.0	0.019	73.5
2	0.010	98.4	0.013	93.7
3	0.016	98.3	0.017	92.5
4	0.008	98.0	0.011	97.5
5	0.011	98.7	0.013	97.1
6	0.010	99.7	0.024	61.7
7	0.027	98.5	0.025	85.5
8	0.007	98.5	0.028	87.8
9	0.014	95.8	0.015	74.2
10	0.007	99.7	0.027	87.4
11	0.007	99.9	0.017	84.3
12	0.007	98.9	0.015	89.6
13	0.011	94.4	0.021	76.5
14	0.009	99.9	0.010	64.3
15	0.023	89.8	0.037	78.3
16	0.030	95.9	0.050	20.7
17	0.031	90.1		0
18	0.008	100	0.019	62.2
19	0.019	86.9	0.035	67.0
20	0.016	97.7		0
21	0.009	97.5	0.030	79.5
22	0.014	95.5	0.032	62.5
23	0.010	99.0	0.009	98.3
Median	0.011	98.3	0.022	78.3
IQR	0.008	3.6	0.014	26.2

**Table 3:** Performance metrics reported in sleep studies.

Study	Sensor	Location	Participants	Methodology	Rate	Performance Metrics
[31]	ACC	Chest	12, AHI<5	Axes fusion	RR	ρ=0.20
[32]	ACC	Wrist	34, AHI<5	Axis selection	RR	ρ=0.88, $T_{C}=48.3\%$
[33]	ACC	Chest	7, AHI<5	Axes fusion	RR HR	ρ=0.92 ρ=0.93
[34]	ACC & GYRO	Wrist	3, AHI<5	Axis selection and rate fusion	HR	MAE=0.95(3.48) min^−1^, $T_{C}=85.9\%$
[35]	ACC	Chest	13, AHI<5	Axes fusion	RR	MAE=1.8(2.2) min^−1^
[41]	ACC	Wrist	182, AHI>5	Axis selection	HR	ρ=0.93, $T_{C}=25.7\%$
[42]	ACC	Chest	11, AHI=5.8(4.2)	Axis selection	RR	MAE=1.67(0.37) min^−1^
Current	ACC & GYRO	Waist	23, AHI=27.5(33.0)	Axes fusion	RR HR	MAE=0.66(0.48) min^−1^, ρ=0.95, $T_{C}=98.3\%$ MAE=1.32(0.84) min^−1^, ρ=0.96, $T_{C}=78.3\%$

Values are denoted as numbers and mean (SD) or median (IQR); ACC: 3D accelerometer; GYRO: 3D gyroscope.

## Data Availability

The data that support the findings of this study are available from G.R. (g.rigas@pdneurotechnology.com) upon reasonable request.

## Abbreviations

The following abbreviations are used in this manuscript:

RR	Respiratory rate
HR	Heart rate
ICU	Intensive care unit
ECG	Electrocardiogram
BCG	Ballistocardiogram
SCG	Seismocardiogram
GCG	Gyrocardiogram
SDB	Sleep-disordered breathing
SNR	Signal-to-noise ratio
PCA	Principal component analysis
IMU	Inertial measurement units
AHI	Apnea–hypopnea index
OSA	Obstructive sleep apnea
PSG	Polysomnography
PD	Parkinson’s disease
MAE	Mean absolute error
IQR	interquartile range
